# Anti-CTLA-4 nanobody as a promising approach in cancer immunotherapy

**DOI:** 10.1038/s41419-023-06391-x

**Published:** 2024-01-08

**Authors:** Mehregan Babamohamadi, Nastaran Mohammadi, Elham Faryadi, Maryam Haddadi, Amirhossein Merati, Farbod Ghobadinezhad, Roshanak Amirian, Zhila Izadi, Jamshid Hadjati

**Affiliations:** 1 Department of Biology, School of Natural Sciences, University of Tabriz, Tabriz, Iran; 2 Stem Cell and Regenerative Medicine Innovation Center, Tehran University of Medical Sciences, Tehran, Iran; 3 USERN Office, Kermanshah University of Medical Sciences, Kermanshah, Iran; 4 Student Research Committee, Kermanshah University of Medical Sciences, Kermanshah, Iran; 5 Department of Immunology, School of Medicine, Kermanshah University of Medical Sciences, Kermanshah, Iran; 6 Department of Medical Laboratory Sciences, School of Paramedical, Kermanshah University of Medical Sciences, Kermanshah, Iran; 7 Pharmaceutical Sciences Research Center, Health Institute, Kermanshah University of Medical Sciences, Kermanshah, Iran; 8 Department of Immunology, School of Medicine, Tehran University of Medical Sciences, Tehran, Iran

**Keywords:** Cancer immunotherapy, Immunosuppression

## Abstract

Cancer is one of the most common diseases and causes of death worldwide. Since common treatment approaches do not yield acceptable results in many patients, developing innovative strategies for effective treatment is necessary. Immunotherapy is one of the promising approaches that has been highly regarded for preventing tumor recurrence and new metastases. Meanwhile, inhibiting immune checkpoints is one of the most attractive methods of cancer immunotherapy. Cytotoxic T lymphocyte-associated protein-4 (CTLA-4) is an essential immune molecule that plays a vital role in cell cycle modulation, regulation of T cell proliferation, and cytokine production. This molecule is classically expressed by stimulated T cells. Inhibition of overexpression of immune checkpoints such as CTLA-4 receptors has been confirmed as an effective strategy. In cancer immunotherapy, immune checkpoint-blocking drugs can be enhanced with nanobodies that target immune checkpoint molecules. Nanobodies are derived from the variable domain of heavy antibody chains. These small protein fragments have evolved entirely without a light chain and can be used as a powerful tool in imaging and treating diseases with their unique structure. They have a low molecular weight, which makes them smaller than conventional antibodies while still being able to bind to specific antigens. In addition to low molecular weight, specific binding to targets, resistance to temperature, pH, and enzymes, high ability to penetrate tumor tissues, and low toxicity make nanobodies an ideal approach to overcome the disadvantages of monoclonal antibody-based immunotherapy. In this article, while reviewing the cellular and molecular functions of CTLA-4, the structure and mechanisms of nanobodies’ activity, and their delivery methods, we will explain the advantages and challenges of using nanobodies, emphasizing immunotherapy treatments based on anti-CTLA-4 nanobodies.

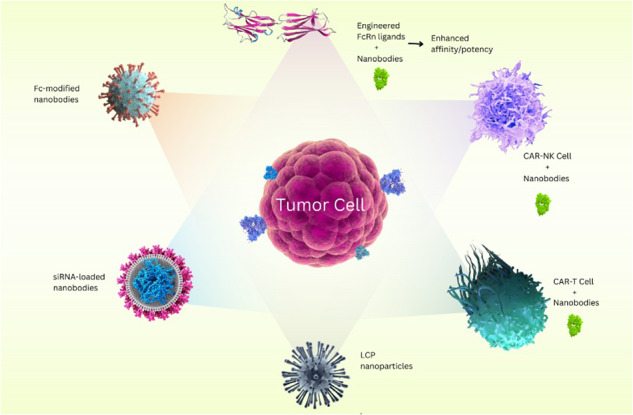

## Facts

- Cytotoxic T lymphocyte-associated protein-4 (CTLA-4) is an essential immune molecule that plays a vital role in cell cycle modulation, regulation of T cell proliferation, and cytokine production.
- Inhibition of overexpression of immune checkpoints such as CTLA-4 receptors has been confirmed as an effective strategy in cancer therapy.
- The “Nanobodies” with small size, low toxicity, low immunogenic potential, high stability (even in the presence of degrading enzymes, low pH, and thermally harsh conditions), solubility, high affinity to the target antigen, ease of production in bacteria, and long shelf-life make them excellent tools to be exploited in solid tumor immunotherapy.
- The developing nanobody-based strategies to inhibit CTLA-4 checkpoint could have promising results in treating hematological and solid tumors.

## Open questions

- Can nanobody-based drugs be successful in cancer treatment and diagnosis?
- Can anti-CTLA-4 nanobodies provide promising results in clinical research?
- What solutions are suggested to deal with the limitations of producing modified nanobodies with Fc-receptors?

## Introduction

Immune checkpoints are a group of inhibitory receptors with vital mechanisms to regulate effector immune cells and prevent them from eliminating normal cells and causing autoimmune diseases [1]. However, this mechanism is exploited by cancer cells to evade the immune system and suppress the effector actions, which can result in immunosurveillance [2]. Natural killer cells and T cells mainly express checkpoint receptors, namely cytotoxic T-lymphocyte-associated protein 4 (CTLA-4), programmed death receptor 1 (PD-1), programmed death-ligand 1 (PD-L1), killer immunoglobulin-like receptors (KIR), lymphocyte-activation gene 3 (LAG-3), and T cell immunoglobulin and mucin domain-containing-3 (TIM-3) [3]. Blocking these inhibitory molecules overcomes the immune escape and promotes an anti-tumor immune response [4]. To reach this goal, several groups focused on developing monoclonal antibodies (mAbs) and small molecules to enhance the immunotherapeutic outcome [5, 6]. In recent years, mAb-based immune checkpoint inhibitors such as anti-CTLA-4, anti-PD-1, and anti-PD-L1 have become hotspots in immunotherapy. Therefore, inhibiting immune checkpoints based on monoclonal antibodies is a primary approach for treating and diagnosing various cancers [7]. A legion of mAbs against these molecules has been approved by the Food and Drug Administration (FDA) after showing favorable outcomes in preclinical studies followed by clinical trials [8]. However, conventional mAb-based immunotherapy faces several drawbacks, including low tissue penetration, high production costs, low binding affinity, and immune-related adverse events [9–11]. Several strategies have been utilized to overcome these hindrances to improve the immunotherapeutic fruition. Among them, the miniaturization of antibodies draws much attention. Synthetic or naturally derived antigen-binding fragments (Fabs, ~50 kDa), variable fragments (Fvs, ~15 kDa), and single-chain variable fragments (scFvs, ~30 kDa) were generated to conquer the flaws of full-length antibodies. These mAb-derived fragments show some benefits, such as faster clearance and improved penetration, but they exhibit some disadvantages, including decreased stability, lower affinity, and difficulties in large-scale production [12]. A unique type of antibody, the heavy-chain antibody (HcAb, ~95 kDa), was tracked down in the blood of camelids and sharks [13, 14]. Despite the lack of light chains and the first constant CH1 domain, HcAbs are fully functional and have antigen-binding affinities similar to conventional mAbs [15]. Interestingly, the specificity of HcAbs is driven by their heavy variable domains (VHHs, also known as single domain antibodies, ~15 kDa), which are considered the smallest naturally derived antigen-binding fragment [9, 16]. The term “nanobodies” was first utilized by the Belgian company Ablynx® concerning their nanometer size (4 nm × 2.5 nm × 3 nm dimensions) [17]. Nanobodies benefit from a myriad of avails, including small size, low toxicity, low immunogenic potential (since VHHs and the human VH framework of family III have more than 80% sequencing identity), stability (even in the presence of degrading enzymes, low pH, and thermally harsh conditions), solubility, high affinity to the target antigen, ease of production in bacteria, and long shelf-life [16, 18, 19]. As mentioned, conventional antibody-based immunotherapies do not exhibit promising results in solid tumors because of their low penetrating ability and large size. Also, other factors, such as the expression of multiple immune molecules, low immunogenicity, low lymphocytic tumor infiltration, lack of IFNg signaling, and hypoxia, play an important role in preventing the effectiveness of conventional antibody-based immunotherapies [20, 21]. Therefore, the ideal size and other properties of nanobodies, which we discuss further, make them excellent tools to be exploited in solid tumor immunotherapy. In addition, CTLA-4 is a well-studied immune checkpoint expressed on activated T cells. The inhibition of CTLA-4 via immune checkpoint inhibitors showed promising results in treating hematological and solid tumors [22, 23]. Herein, we review the current studies on developing nanobody-based strategies to improve CTLA-4-based immunotherapy and immunoimaging.

## Structure and expression of CTLA-4

### Structure of CTLA-4

**Fig. 1:**
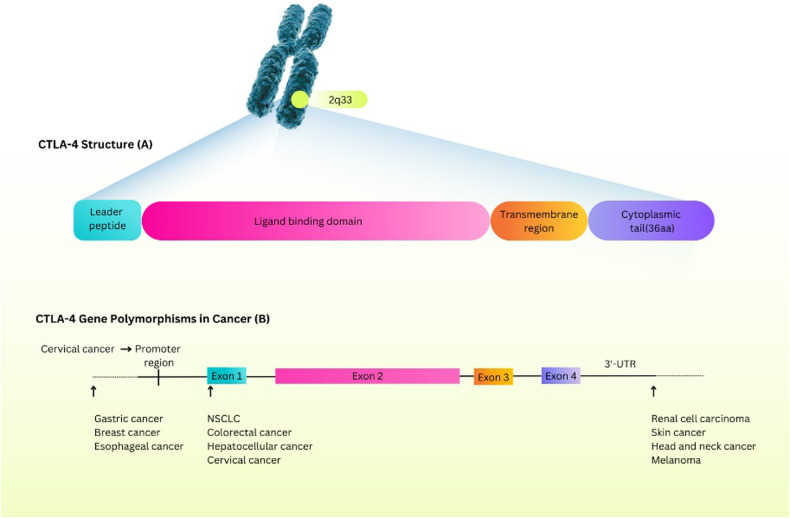
Structure and expression of CTLA-4. The locus of the CTLA-4 gene on chromosome 2 and the constituent parts of the expressed protein of the CTLA-4 gene (**A**). The role of CTLA-4 gene polymorphisms in different cancers (**B**). CTLA-4 protein consists of a leader peptide and three domains: a ligand-binding region, a transmembrane region, and a cytoplasmic tail. As shown in the picture, these parts correspond to exon 1, exon 2, exon 3, and exon 4 in the CTLA-4 gene, respectively. CTLA-4 Cytotoxic T-Lymphocyte Associated Protein 4, AA Amino Acids, NSCLC Non-Small-Cell Lung Cancer, 3′-UTR 3′-Untranslated Regions.

CTLA-4 (CD152) is one of the immune checkpoint molecules with a molecular weight of 25 kDa [24]. This molecule is primarily expressed in activated T lymphocytes and regulatory T lymphocytes (Tregs) that prohibit the activation of T cells [25–27]. CTLA-4 comprises four essential parts: a leader peptide and three domains. The first domain interacts with ligands, and the second and third domains act as transmembrane and cytoplasmic domains [24]. In addition, these domains are associated with exons 2, 3, and 4, respectively [28]. The CTLA-4 gene is located at 2q33, and several Single Nucleotide Polymorphisms (SNPs) related to this gene have been detected. CTLA-4 gene polymorphisms are associated with susceptibility to different malignancies (Fig. 1) [28]. These SNPs are effective in gene expression and protein functions [29]. Several costimulatory receptors adjust T cell responses positively and negatively [30]. CTLA-4 and CD28 are equivalent in structure [31] and compete in binding to B7 family members. CTLA-4 has higher avidity to its ligands [32]. CD28 binds to B7.1 (CD80) and B7.2 (CD86), which are highly expressed by antigen-presenting cells (APCs). However, in the presence of CTLA-4, these ligands have a greater affinity for binding to CTLA-4 [5, 33]. It will be fully explained in the following sections.

Although CTLA-4 and CD28 molecules have the same ability to bind to B7 family molecules, they mediate opposite effects on T cell function [34]. For example, CTLA-4 reduces the response of T cells in different ways, while CD28 increases the proliferation of these cells [35–38].

### Expression of CTLA-4

In addition to being expressed in T lymphocytes, CTLA-4 can be expressed in other cells, such as B cells, granulocytes, and fibroblasts [39–41]. Furthermore, resident immune cells in the tumor microenvironment express higher levels of CTLA-4; Hence, CTLA-4 can be found in malignancies caused by immunosurveillance [42, 43].

In general, CTLA-4 is expressed by Treg cells. However, stimulation of conventional T cells leads to upregulating CTLA-4, in which the nuclear factor of activated T cells (NFAT) is influential [44]. Before the activation of conventional T cells, insignificant amounts of CTLA-4 are expressed in the intracellular parts, but after activation, the expression of this molecule on the cell surface increases dramatically [33, 45]. Foxp3 is a significant transcription factor in the activation of Tregs that promote CTLA-4 expression [46]. CTLA-4 is also expressed by pituitary cells, particularly cells that secrete TSH and prolactin [47].

## The cellular and molecular functions of CTLA-4

### The biological function of CTLA-4

**Fig. 2:**
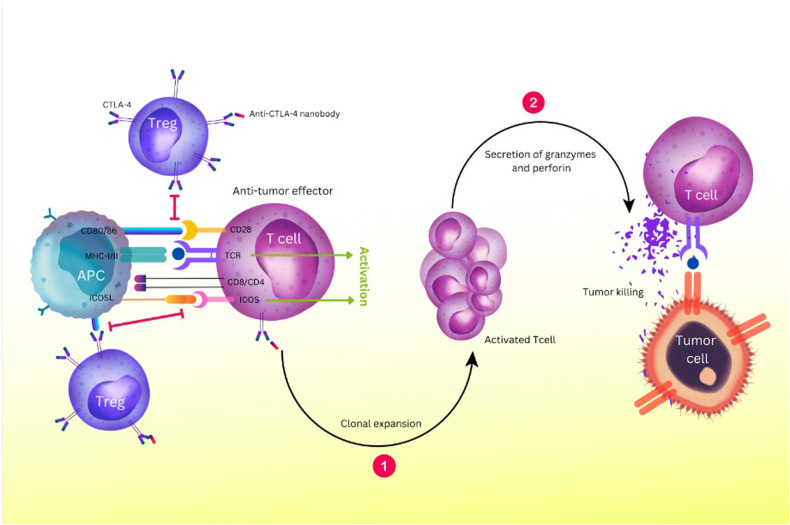
CTLA-4 is a regulatory molecule highly expressed in Tregs and suppresses the function of tumor-reactive T cells. Blocking the CTLA-4 axis with anti-CTLA-4 nanobodies inhibits this binding. Instead, it improves the activity of anti-tumor T cells by increasing the activation and proliferation of T cells and the increase of memory cells. This causes more T cells to bind to tumor antigens presented by MHC molecules. It also causes the upregulation of ICOS to stimulate the proliferation of T cells, produce cytokines, and ultimately promote anti-tumor responses. Furthermore, it produces cytolytic mediators such as perforin and granzyme, increasing tumor killing. MHC Major Histocompatibility Complex, ICOS Inducible T cell Co-Stimulator, ICOSL ICOS Ligand, Treg Regulatory T cell, APC Antigen-Presenting Cell, TCR T-cell receptor.

The biological activity of CTLA-4 and its expression is crucial for controlling T cell activation. Serine/threonine phosphatase family members, including PP2A and PP6, interact with CTLA-4 to block CD28 signaling downstream pathways [48]. Treg cell-intrinsic signaling pathway plays a part in activating CTLA-4’s cell-extrinsic suppressive activity on activated T cells [49]. It has also been proposed that CTLA-4’s binding to CD80/86 causes dendritic cells (DCs) to produce the enzyme indoleamine 2, 3-dioxygenase (IDO) that breaks down tryptophan [50]. Lack of tryptophan causes the cell cycle to arrest in the mid-G1 phase and reduces T cell responses [51]. Additionally, CTLA-4 reduces T follicular regulatory (Tfr) and T follicular helper (Tfh) cell expansion, which is one way that CTLA-4 controls B cell responses [52]. By inhibiting effector and proliferative processes, CTLA-4 on Treg cells may control memory CD8^+^ T cells’ inactivity (Fig. 2) [53].

### Comparison of the Interaction between CTLA-4 and CD28 with Ligands about CD80 and CD86

**Fig. 3:**
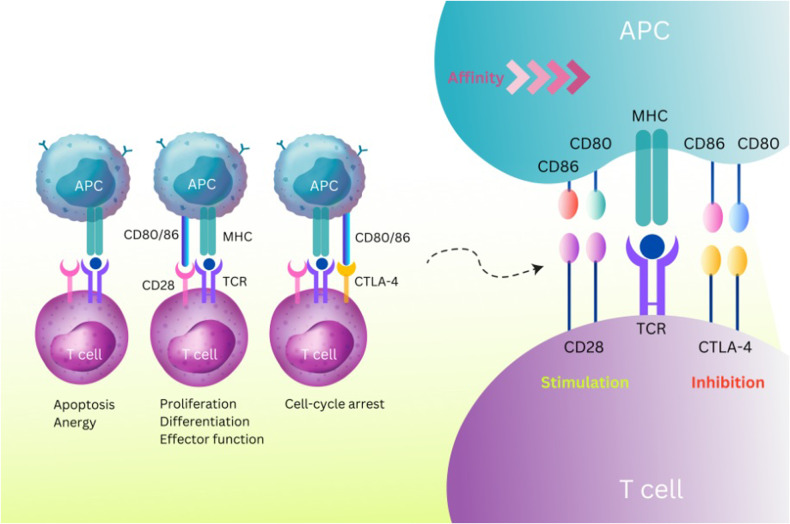
CTLA-4 Interaction with CD80 and CD86 Compared with CD28. CTLA-4 competes with the stimulatory molecule CD28 for binding to CD80/86 ligands on the surface of APCs, for which it has a higher affinity and avidity. Following the recognition of the MHC-peptide complex by the TCR (signal 1) and B7 (Cd80/86) by the co-stimulatory receptor CD28 (signal2), T cell activation, proliferation, and differentiation occur. Conversely, when CD28 signaling is absent, T cells cannot respond and either undergo apoptosis or become anergic. Also, following T cell activation and CTLA-4 overexpression, CTLA-4 signals cause cell-cycle arrest and stop T cell activation. CTLA-4 and its homolog CD28 are critical T cell proteins that play opposing roles in T cell activation. They bind to the same CD80 and CD86 ligands found on antigen-presenting cells (APCs). However, CTLA-4 has higher affinity and avidity for ligands than CD28, indicating a significant biological divergence.

CTLA-4 and CD28 are homologous immunoglobulin superfamily receptors that control numerous facets of T cell immune regulation [54]. Two ligands, CD80 and CD86, are shared by the CTLA-4 and CD28 receptors. CD86 is a monomeric lower-affinity ligand for both receptors, and CD80 is a dimeric high-affinity ligand. Compared to CD28, CTLA-4 binds with both ligands more avidly and with higher affinity [54, 55]. Although CTLA-4 and CD28 have similar ligands, their expression pattern differs [48]. CD28 is expressed on the surface of the most naïve CD4 and CD8 T cells and is the central costimulatory molecule in initial T cell activation [56]. On the other hand, CTLA-4 is expressed on activated T cells, particularly regulatory T cells (Tregs), and induces inhibitory signals in T cells, leading to the termination of T cell responses (Fig. 3) [57]. The regulation of APC activation and function by depletion of CD80 and CD86 is a cell-extrinsic mechanism of CTLA-4 to prevent CD28 co-stimulation (Fig. 3) [58, 59]. The molecular basis for the cell-extrinsic mechanism of CTLA-4 is the trans-endocytosis of two ligands (CD80 and CD86) from the surface of the APC to inside the T cell [59].

Another potential mechanism for CTLA-4’s suppression of T cells could be competition with CD28 in binding to B7 molecules [60]. Generally, the balance of stimulatory and inhibitory signals in controlling inflammation is mediated by CD28 and CTLA-4, which dominate the outcome of T cell responses [61].

### Interaction of CTLA-4 with Phosphatidylinositol 3-Kinase (PI3K)

During activation of T cells, the Src-family protein tyrosine kinases (PTKs), Lck and Fyn phosphorylate CD3 and TCR. For example, ZAP-70, a Syk-family PTK, is essential for signaling via the TCR through its tandem Src-homology 2 domains and is subsequently phosphorylated by Lck and Fyn [62, 63]. SLP-76 and linker of activated T cells (LAT) are two adaptor proteins that activated ZAP-70 must phosphorylate for T cells to develop and become activated [62]. Through its interaction with SLP-76 and LAT, the SH2 domain of Gads plays a crucial part in the T cell signaling cascade [64]. Phosphatidylinositol-4, 5-bisphosphate (PIP2) is hydrolyzed by PLC-1 to create inositol-1, 4, 5-triphosphate (IP3) and diacylglycerol. IP3 causes a prolonged increase in intracellular calcium. At the same time, diacylglycerol encourages the activation of protein kinase C (PKC) [63]. PKC then activates the small GTPase Ras, and Ras, in turn, triggers several signaling molecules, such as the mitogen-activated protein kinases (MAPK) [65]. Cellular activities, such as metabolism, motility, gene expression, and programmed cell death, are regulated by MAPKs via phosphorylating their target protein substrate [66]. An extended rise in intracellular calcium activates the phosphatase, such as calcineurin. As a result, calcineurin causes nuclear transcription factors (NFAT) to translocate into the nucleus of the cytoplasm. Members of the NFAT family, respectively, enhance the expression of different immune responses or essential genes in T cells [67]. CTLA-4 on the surface of T cells could suppress both TCR and TCR-CD28 expression. This suppression was followed by a blockade when the linker for activation of T cells (LAT) was present in purified membrane rafts. As a result, CTLA-4 targets the expression of these rafts [68]. Src kinases promote CTLA-4 cytoplasmic phosphorylation and PI-3 kinase recruitment [69]. The relation between CTLA-4 and tyrosine phosphatase SHP-2 is indirect and may be mediated through PI3-kinase/SHP-2 binding [70].

### Interaction of CTLA-4 with AP-1 and AP-2 complexes

FoxP3^+^ Treg cells or activated conventional T cells are the primary sources of intracellular vesicles containing CTLA-4. Its endocytosis from the plasma membrane is responsible for its intracellular localization, which results in around 90% of CTLA-4 being intracellular [33, 45]. More than 80% of surface CTLA-4 is internalized within five minutes due to the fast endocytosis of CTLA-4 [71]. After internalization, CTLA-4 molecules return to the plasma membrane or are degraded in lysosomal compartments [54]. It is believed that CTLA-4 is transferred from an intracellular compartment to the cell surface by T cell activation in a way that may be related to the amount of T cell receptor signaling [71].

The heterotetrameric adapter protein AP-2 mediates the removal of CTLA-4 from the cell surface. This protein performs this function through clathrin-dependent internalization [72]. For AP-2 binding, CTLA-4 has a Gly-Val-Tyr-Val-Lys-Met (GVYVKM) motif [73]. The phosphorylation of a smaller region within the same motif (YVKM) in CTLA-4’s cytoplasmic tail regulates AP-2 binding. Tyrosine-containing motif YVKM in the cytoplasmic domain of CTLA-4 interacts with the medium-chain (μ2) of AP-2 when it is not phosphorylated. At the same time, The phosphorylation of the YVKM motif prevents AP-2 from binding to CTLA-4 [54, 72].

Additionally, the AP-1 complex interacts with a GVYVKM motif in CTLA-4 in the Golgi compartment, keeping the intracellular receptor steady. The AP-1 complex shares structural similarities with the AP-2 complex but is found in the TGN and lysosomes. CTLA-4-AP-1 interaction mediates transport from the TGN to endosomal and lysosomal compartments for degradation. As a result, AP-1 and AP-2 in T cells provide ways to control CTLA-4 intracellular levels [61, 72].

## Structure of nanobodies

**Fig. 4:**
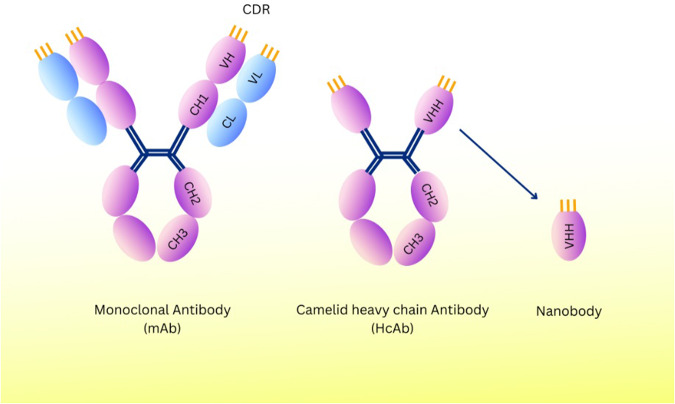
Schematic representation of monoclonal antibodies (mAb) and nanobodies generated from camelid heavy chain antibodies (HCAbs). Nanobodies (or VHH antibodies) are the antigen-binding fragments of heavy chain-only antibodies. They have a lower molecular weight than mAb, lacking the light chain and the heavy chain’s CH1 domain. Compared to the VH-VL domains in mAbs, the CDR3 loop of the VHH/Nanobody is significantly longer, giving antigen affinity and access to concealed epitopes. mAb Monoclonal Antibody, CH Heavy Chain Constant Region, CL Light Chain Constant Region, VH Variable Regions of the Heavy, VL Variable Regions of the Light, CDR Complementarity-Determining Regions, VHH Heavy-Chain Variable Domain, HcAb Heavy-chain Antibody.

Nanobodies (Nb) are single-variable domains of heavy-chain antibodies (hcAbs), which are naturally derived from llamas and other camelids [74]. Variable antigen-binding domains (VHH) have a molecular mass of 15 kDa, whereas heavy-chain antibodies have a 95 kDa molecular mass. These VHHs have diameters of 3 nm, 4 nm, and 2.5 nm in their prolate shape (Fig. 4) [75]. Conserved framework regions (FRs) and antigen-binding sites of hypervariable regions, known as complementarity-determining regions (CDR), make up the structure of a nanobody. Nanobodies’ CDR3 loop, which has a mean of 18 amino acid residues and a finger-like shape, allows them to bind antigens, and better antigen interaction results from longer CDR3 sequences [76]. In addition, due to their hydrophilic surface and the fact that they do not bind light chains, Nbs can be easily combined into dimers and multimers [77].

## Targets and applications of nanobodies

**Fig. 5:**
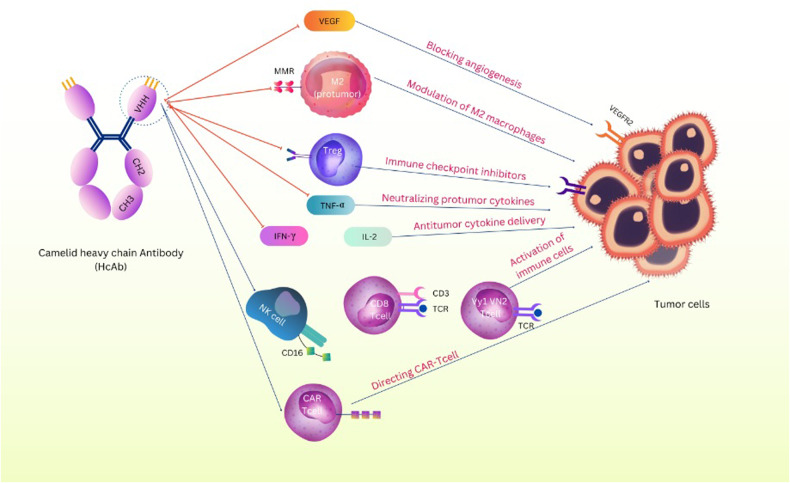
Mechanism of nanobodies in tumor targeting. The mechanisms by which nanobodies target the tumor microenvironment include: Blocking the VEGF/VEGFR pathway for suppressing angiogenesis, Modulation of M2 macrophages by targeting the MMR, Blocking immune checkpoint molecules by nanobodies that enhance the anti-tumor T cell effector activity, Targeting the Tumor Environment Cytokines and Chemokines as critical modulators of immune cell states of activation, Activation of immune cells such as NK cells, γδ T cells, and CD8^+^ T cells, Directing CAR-T Cell. VEGF Vascular Endothelial Growth Factor, MMR Mannose Macrophage Receptor, TNF-α Tumor Necrosis Factor-α, IFN-γ Interferon- γ, IL-2 Interleukin 2, NK Cell Natural Killer Cell, VEGFR VEGF Receptor, CAR-T Cell Chimeric Antigen Receptor T Cell.

Numerous studies on nanobodies have been conducted about immunotherapy and targeted tumor therapy. Therefore, the following categories can be used to classify the various cancer prevention methods that incorporate nanobodies (Fig. 5).

### Blocking angiogenesis

Nanobodies are promising to fight tumor angiogenesis, progression, and metastasis. Since vascular endothelial growth factor (VEGF) and its receptors are known stimulants, they are excellent targets for suppression. A nanobody against the VEGF receptor-2 (VEGFR2) showed that it prevented the development of capillaries [78]. Also, a nanobody in EGFR targeting regulation has been developed. This nanobody is combined with a bi-paratopic anti-EGFR nanobody known as CONAN-1. The CONAN-1 inhibits tumor expansion less effectively than the EGFR-targeting antibody (cetuximab). The lack of Fc on the nanobody structure may cause its lower potency, indicating a more significant role for immune cells in destroying EGFR-expressing tumors [79].

### Modulation of M2-macrophages

A significant factor in the development of tumors is the M1/M2 macrophage pattern [80]. M1 macrophages produce reactive nitrogen species, reactive oxygen species, and pro-inflammatory cytokines such as IL-6 and TNF-α to inhibit tumor growth. This is while M2 macrophages secrete matrix-degrading enzymes, angiogenic factors, and anti-inflammatory cytokines such as IL-10 and TGF-β, thereby creating an immunosuppressive environment and favoring tumor growth [81, 82]. In addition to the above, when the MMR (mannose macrophage receptor) specific nanobody is combined with pro-apoptotic proteins such as second-mitochondria derived activator of caspases, it has shown precise targeting to and modulation of M2-macrophages [83].

### Blocking immune checkpoints

Immune checkpoint molecules such as PD-1/PD-L1 and CTLA-4 are crucial for developing tumors and immune responses [84]. For example, a study by Wan R et al. in melanoma-bearing mice (B16) after treatment with the nanobody “NB16” that targets CTLA-4 showed a significant reduction in tumor growth and increased survival time [85]. Additionally, increased anti-tumor effects can be achieved by combining the PD-L1 nanobody with avelumab, the mAb counterpart of PD-L1 [86].

One of the most crucial innate checkpoints is the SIRPα-CD47 axis. The ubiquitous self-marker CD47 serves as a warning to other cells not to eat it. Through contact with the immunological checkpoint receptor SIRPα, it suppresses macrophage phagocytosis. Unfortunately, many types of cancer disrupt this pathway by overexpressing CD47 to defend themselves from immunological responses [87]. As a result, anti-CD47 nanobodies were created to revive phagocytosis mediated by macrophages. For instance, tumor cell phagocytosis by macrophages was markedly enhanced by an anti-mouse CD47 nanobody [88].

### Delivering anti-tumor cytokines

The combination of nanobodies in cytokine treatments shows a new level of effectiveness. TNF-α, as one of the main inflammatory cytokines, suppresses immune response by promoting the development of immune-suppressive regulatory T cells and myeloid cells in the tumor microenvironment. Findings show that TNF-targeted nanobodies can reduce lung metastases when combined with the anti-mitotic drug paclitaxel [89, 90].

Also, combining an anti-PD-L1 nanobody with either IL-2 or IFN was effective in vivo in pancreatic cancers resistant to therapy (IFN) [91]. In addition, an anti-CEA nanobody fused with IL-12 also demonstrated increased anti-tumor activity [92].

### Activation of immune cells (CD8^+^ T, NK, and γδ T-Cells)

Cytokines are crucial in signaling the activation of CD8^+^ T cells, but they only serve as co-stimuli. Without TCR activation and subsequent CD3ζ signaling, CD8^+^ T cells cannot differentiate into CTLs. As a result, strategies have been created to mimic CD3ζ signaling without MHC-I: peptide-mediated TCR triggering. These depend on agonistic antibodies or cross-linked antibody fragments binding to CD3 to function. To ensure cross-linking and subsequent T cell activation, nanobodies that bind EGFR on tumor cells have been combined with anti-CD3 scFvs in their monovalent or trimer forms [93, 94].

Without a costimulatory signal, the NK cell-expressed CD16 (FcRIII) receptor stimulates NK cell proliferation through the PI3K/MAPK pathways [95]. The C21 is a nanobody for CD16 that stimulates the production of IL-2 and IFN by NK cells. This nanobody causes this after polymerization by biotinylation of sdAb and exposure to streptavidin [96].

Stimulation of γδ T cells, especially Vγ9Vδ2 T cells, promotes anti-tumor effects [89]. Therefore, the expansion and activation of these cells seem to be a potential therapeutic approach [97]. For example, a bispecific antibody made of anti-EGFR and anti-Vγ9Vδ2 TCR nanobodies can activate Vγ9Vδ2 T cells. However, according to the findings, this nanobody causes toxicity against EGFR^+^ tumor cell lines [98].

### Cancer vaccines

Cancer vaccines are designed to stimulate CTLs activation and proliferation [99]. They can develop an immunological memory that prepares them to respond when new cancer cells with the same characteristics appear. In addition, they can selectively identify and destroy cancer cells regardless of their location. To activate CTLs, tumor antigens must be presented in MHC-I molecules and co-stimulated by professional APCs [100, 101]. In order to convey tumor antigens or enhance the APC’s ability to present antigens, nanobodies have been connected to several technological platforms. Targeting APCs increases the vaccine’s effectiveness and minimizes potential side effects [89]. Multiple proteins expressed on the surface of APCs, including CD11b, MHC-II, 161, CD1d, Clec9a, and PD-L2, have been targeted with nanobodies [86, 102]. The anti-CD11b nanobody produced the most significant activation of CD8^+^ T cells [103]. Crowley et al. also created nanobody-conjugated peptide vaccines that targeted MHC-II for APC delivery and showed improved vaccine-mediated CD8^+^ T cell activation [104]. In addition, many nanobodies target APCs whose antigens have yet to be discovered, such as DC1.8, DC2.1, and R3_13 nanobodies [105, 106].

## Anti-CTLA-4 nanobodies in cancer treatment

### The advantage of using nanobodies in cancer immunotherapy

Many studies on cancer immunotherapy have been conducted on monoclonal antibodies (mAbs). However, some reasons reduce the penetration of mAbs into solid tumors, such as large size, low stability, and high half-life during molecular imaging [107]. Therefore, nanobodies have been developed to overcome the limitations above. Various reasons, such as their tiny size, stability, specificity, high affinity, and simple creation procedure, have candidate nanobodies for therapeutic and imaging purposes [108]. In this section, we go over the benefits of nanobodies as they relate to their use in treating malignancies (Table 1).

**Table 1:** Essential characteristics of nanobodies in different dimensions.

Nanobodies	Property	Refs.
History	Discovered in 1992	[211]
	Found in camels and llamas	[212]
Structures	Small pieces of protein	[213]
	Derived from the heavy chain and variable antibodies (VHH)	[214]
	In the absence of the light chain, they have evolved completely	[215]
	Lack of the Fc domain	[216]
	Have a hydrophilic side	[217]
Advantages	Having the good characteristics of conventional monoclonal antibodies	[218]
	Use as pharmaceutical molecules	[219]
	High solubility	[217]
	More flexibility	[220]
	Small size	[217]
	Fast and easy penetration into tissues	[158]
	Identification of many antigens	[221]
	High antigen binding capacity	[222]
	High resistance to changes in temperature and pH	[223]
	Significant resistance against enzymes (Especially trypsin and pepsin)	[223]
	Easy to make and adjust	[216]
	Including multi-specific, multivalent, and bi-paratopic constructs	[224]
	Low viscosity	[224]
	Enabling multiple routes	[224]
	Application in a wide range of diseases (Immune, bone, blood, and neurological disorders)	[225]
	Reasonable cost to build	[226]
	Good expression efficiency	[227]
	Application in cancer diagnosis and treatment	[174]
	Have low toxicity	[228]
	Have easier handling	[220]
	Use as chromobodies	[229]
	Use as protein purification	[230]
	Use in non-invasive imaging	[231]
Limitations	Short half-life (A few hours)	[232]
	Rapid clearance from the blood	[232]
	Slow drug release	[233]

*VHH* variable heavy domain of heavy chain, *Fc* fragment crystallizable.

Nanobody has low molecular weight and small size (15 KD) compared to traditional antibody, making it easy for them to penetrate the tumor tissue. Also, some nanobodies can cross the blood-brain barrier (BBB). Additionally, in molecular imaging, the small size of nanobodies leads to fast tumor accumulation and rapid clearance from blood circulation. Small size is one of the significant physiochemical features of a nanobody (Table 1) [86, 109]. Also, nanobodies have hydrophilic regions and considerably longer CDR3 than human VH domains, contributing to their high affinity and specificity for their target antigens in cancer therapy [86, 110]. Nanobodies can be refolded after thermal denaturation [111, 112]. According to recent studies, the thermal denaturation of nanobodies may be irreversible. The results of these studies show the thermal resistance of nanobodies. Furthermore, these studies have shown that nano-object accumulation decreases following thermal degradation. This is due to the additional disulfide bonds [111, 113–116]. Two mechanisms have explained the cause of this issue. First, nanobodies with two disulfide bonds may increase kinetic stability compared to a single bond. This results in a folding equilibrium with less tendency for nanobodies to aggregate [111, 117, 118]. Second, disulfide bridges were suggested to protect native proteins, implying a more direct interference of disulfide bonds with the nanobodies’ aggregation [111, 119, 120].

As previously stated, nanobodies play a critical role in tumor identification by molecular imaging. The most important reasons are their better penetration into the tumor and longer serum half-life compared to antibodies. So far, no clinical trial (Phase III) has been conducted to target CTLA-4 by nanobodies. However, the most cutting-edge nanobody probes are currently being tested in clinical settings and target HER2 [86]. In addition, nanobody probes have been used for immunological checkpoints such as CTLA-4 and PD-L1. These probes can have nuclear imaging with a high T/B ratio [121–123]. Therefore, In addition to identifying the tumor antigen profile, nanobodies also control Ag penetration into immune cells and activation levels [86]. Blocking tumor angiogenesis is another potential role of nanobody in cancer treatment. For instance, VEGF, the critical mediator of angiogenesis, plays a significant role in tumor growth and metastasis formation. Therefore, the VEGF-VEGFR system is a potential target for monovalent and bivalent nanobodies in tumor angiogenesis [86, 124, 125].

Based on this, nanobodies have shown significant results in preclinical studies. However, due to the need for clinical trial studies, more research is needed to investigate the suppressive effects of nanobodies on CTLA-4.

### Targeting immune checkpoints using nanobodies

Checkpoint inhibitory therapies are a powerful clinical tool in cancer treatment. FDA approvals and ongoing clinical development highlight the enormous potential of checkpoint inhibitors as anti-cancer drugs. However, the occurrence of side effects is a significant obstacle to using checkpoint inhibitors as systemic therapies. Hence, methods of sustained and tumor-targeted delivery of checkpoint inhibitors became of interest. These methods will likely improve efficacy and reduce side effects [126]. One of these methods is the use of engineered nanobodies. The importance of nanobodies for cancer treatment and diagnosis has been proven. They are now used as the first line of treatment for some cancers. In addition, nanobodies are considered for targeted drug delivery and radioisotopes due to their unique properties and ease of production [127]. Some therapies use nanobodies to bind to immune checkpoints, such as CTLA-4, to enhance the anti-tumor immune response. CTLA-4 is a negative regulator of T cell activation. Hence, it is a target for cancer immunotherapy [5]. For example, an anti-CTLA-4 nanobody was developed during recent preclinical research for melanoma treatment in-vitro. Mice treated with an anti-CTLA-4 nanobody (Nb16) showed increased survival and delayed melanoma growth [128]. Notably, the combined blockade of PD-1/PD-L1 and CTLA-4-negative stimulatory pathways allows tumor-specific T cells to continue to perform practical functions. Based on the findings, in B16 melanoma tumors, combined PD-1 and CTLA-4 blockade have better results than CTLA-4 blockade alone [129]. In another study, researchers selected high-quality anti-CTLA-4 nanobodies from a camel immune library using phage display technology. Then, four positive colonies in the CDR3 region were classified based on the sequence of amino acids. These nanobodies, which recognized unique epitopes on CTLA-4, showed high binding rates when applied to PHA-stimulated human T cells. The data of this study indicate that anti-CTLA-4 nanobodies are effective in treating tumors [85].

### Suppression of CTLA-4 by H11 nanobody

This section compares the effect of CTLA-4 blockade on cancer treatment by the antibody ipilimumab and the H11 nanobody. In cancer therapy, two factors play an essential role in generating an effective Anti-CTLA-4 antibody response; one is increasing the expression of CTLA-4 on tumor-associated Treg, and the other is increasing the expression of FcγR on tumor-associated macrophages. Increased expression of FcγR is vital in creating a response against the tumor because it facilitates phagocytosis by macrophages [121, 130].

In vivo and in vitro studies on anti-CTLA-4 nanobodies have shown conflicting results. In a study, an H11 nanobody was injected instead of an antibody in a mouse model. This was done to understand the anti-tumor significance of FC. H11 nanobody, which does not have FC and is monovalent, binds with high affinity to mouse CTLA-4 [121, 130, 131].

Furthermore, this nanobody prevented the interaction between CTLA-4 and its ligand [121, 130, 131]. However, an in vivo study has shown that H11 VHH is the least effective when administered as a monomeric antibody fragment (without FC) because it does not affect Treg depletion [121, 131, 132]. Therefore, to increase the anti-tumor function of the H11 nanobody and considering that FC plays an essential role in cancer treatment, H11 is conjugated to mouse IgG2a constant region through fusion or ligation. H11-IgG2a conjugation restores anti-tumor response compared to nanobody alone [121, 133].

Another study examined how the absence of FC fragments in the H11 nanobody and ipilimumab antibody has affected their anti-cancer response [134]. Contrary to previous studies that suggested FC is necessary for the anti-tumor activity [121, 132, 135], another study revealed that anti-CTLA-4 therapy, even without FC, still has robust anti-tumor efficacy [134]. In this study, H11 nanobody was coupled with anti-serum albumin VHH, also known as H11-HLE, having a higher half-life than H11 alone. Therefore, the H11 nanobody, despite lacking FC, has an increased half-life and more anti-tumor properties; however, it does not affect the binding affinity of H11 to CTLA-4 of murine [134]. Although it has been demonstrated that longer half-life extended H11 (H11-HLE) administration has a potent anti-tumor impact in murine models, treatment with H11 alone has a weak anti-tumor effect. Based on the findings, H11-HLE has a superior therapeutic effect compared to anti-mCTLA-4 mIgG2b [134]. As previously reported, besides the H11-HLE nanobody lacking FC, Ipi-LALAPG, another kind of ipilimumab Ab lacking FC, can trigger anti-tumor responses when Treg is not suppressed [136]. Also, another noteworthy point is that starting potent anti-tumor responses by nanobody and antibody-free-FC has indicated these responses are generated independently of FC [134].

According to one study, FC plays a crucial function in lowering the frequency of Treg and increasing anti-tumor activity by conjugating H11 with the murine IgG2a FC [121]. However, another research has found the absence of FC necessary and sufficient to create an anti-tumor response. In this study, H11, with a long half-life, H11-HLE, prevents the interaction of CTLA-4 and its ligand [134].

Anti-CTLA-4 antibodies have side effects such as colitis development. However, anti-CTLA-4 nanobodies have greatly diminished the damage from antibodies and resistance to steroid therapy [137–140]. Therefore, more extensive studies are needed to clarify the conflicting data about the role of FC in stimulating cancer immunotherapy responses.

### CAR-T cell-based nanobodies to block CTLA-4

Chimeric antigen receptors (CARs) are used for immune cell therapies with clinical efficacy. Usually, the structure of these recombinant receptors consists of an antigen-binding domain, a transmembrane domain, and a cytoplasmic domain. These receptors guide immune cells to identify and target molecules on the surface of tumor cells [141, 142]. The CAR structure attacks tumor cells through an antibody-antigen interaction. This genetically engineered molecule usually targets CD19 membrane proteins [143, 144]. This method is the most potent anti-tumor agent based on cell therapy, used in treating cancers, especially blood malignancies [145]. However, there are significant limitations in targeting solid malignancies, including heterogeneous antigens in solid tumors, tumor immunosuppressive microenvironment, cytokines, and immunosuppressive checkpoint molecules by this therapeutic approach [146]. Hence, new therapeutic strategies based on combined treatments of CAR-T cells with other immunotherapy methods or small molecule drugs have been considered to enhance CAR-T cell therapy [146]. The findings show that the nanobodies-based CAR structure has effectively targeted specific tumors. CAR-T or CAR-NK cells based on nanobodies have shown anti-tumor effects in-vitro and in-vivo. Nanobodies have been used as CAR-T antigen binding domains due to their small size, optimal stability, high affinity, and extensive fabrication [142]. According to the research, CAR-T cells of the second-generation nanobodies have more than 50% positive expression. In addition, these cells secrete IFN-γ and IL-2 and increase cytotoxicity in VEGFR2-expressing cells. Nanobodies are also considered attractive modules for three and four-generation CARs [17, 146]. It is worth noting that bivalent nanobodies in bi-specific CAR-T cells can identify two target factors at the same time and reduce tumor escape [147, 148]. Another study discussed the combination of two nanobodies, one similar to TCR-CAR-T nanobodies and the other targeting immune checkpoint inhibitors [146]. This was due to address the tumor microenvironment, increasing the longevity of CAR-T cells, and suppressing tumor growth [149]. These approaches are suitable for improving the nanobody-based antigen binding domain in CARs directed to the MHC complex with TCR specificity [146]. A study on the role of CTLA-4 with CD28 CD19-specific CAR-T cells showed that the downregulation of CTLA-4 by shRNA had no significant effect on CAR-T cells. However, CD19-specific CAR-T cells co-expressing CD80 showed a considerable increase in anti-tumor properties after CTLA-4 was knocked-down [150].

In another study, CAR-T cells secreting anti-PD-L1 or anti-CTLA-4 nanobodies are more stable. In other words, fusion CAR-T cells secreting PD-L1 VHH- and CTLA-4 VHH-Fc leads to better results and significantly contributes to the persistence and survival of CAR-T cells in the body [148].

### siRNA-based Anti-CTLA-4 nanobodies

RNAs are a suitable option for treating diseases, including cancers, due to their ability to regulate protein concentration. Therefore, it is essential to develop safe and effective strategies to help RNAs realize their clinical potential [151]. One of the cancer immunotherapy methods that advances have recently accompanied is to reach the target tissue using biomaterials. Based on immune engineering, this method can increase the efficiency and safety of current cancer treatment methods [152]. Small interfering RNA (siRNA) can be used to develop biomaterials and enhance immune-related cancer gene therapy. siRNAs can play a role in target gene expression and silence a specific gene sequence by inducing the degradation of messenger RNA (mRNA) and thus inhibiting target protein production [152, 153]. Based on the findings, non-invasive methods for siRNA delivery have advantages such as increased stability, targeted delivery, and improved permeability, which have brought promising results in skin cancers [154]. Anti-EGFR nanobodies are ideal tools for the targeted delivery of siRNAs. In this method, a specific site of siRNA is attached to a C-terminal cysteine residue on the engineered nanobody. These siRNA-conjugated nanobodies (Nb-siRNA) retain their binding to EGFR and enter EGFR-positive cells through receptor-mediated endocytosis. Nb-siRNAs are active in vitro and cause mRNA cleavage in the target cell line [155]. According to one study, mannose-modified PEG NPs deliver tumor antigen and adjuvant to dendritic cells and induce a potent, systemic immune response regardless of the presence or stage of the tumor in the host [156]. The researchers enhanced the efficacy of the LCP vaccine by silencing TGF-β in tumor cells, where delivery of siRNA using LPH NP resulted in a 50% reduction of TGF-β in the tumor microenvironment. Depletion of TGF-β increases vaccine efficacy and inhibits tumor growth by 52%. This leads to an increase in tumor-infiltrating CD8^+^ T cells and a decrease in regulatory T cells. This therapy provides a flexible and powerful platform to study the mechanism and develop a cancer immune strategy [156]. For example, in a study, a cationic lipid-coated PEG-PLA NP was developed to deliver CTLA-4 siRNA (siCTLA-4) to T cells in a B16 mouse melanoma model. This research aimed to activate and multiply T cells in vitro and in vivo. This study showed the internalization of systemic siCTLA-4-NPs administered by tumor-infiltrating CD4^+^ and CD8^+^ T cells and increased anti-tumor activity following an increase in the ratio of CD8^+^ T cells to regulatory T cells [152, 157].

### Anti-CTLA-4 nanobodies modified with Fc-Receptors (FcRs)

Nanobodies have many potential advantages over conventional antibodies and are used to develop new cancer treatment strategies for these reasons (Table 1) [158]. Nevertheless, it should be noted that nanobodies do not have the Fc region of a specific antibody. This region has many functions that are considered essential for immunotherapy. Complement-dependent cytotoxicity (CDC) and antibody-dependent cytotoxicity can be mentioned among the critical Fc-dependent functions [159]. The small size of nanobodies can be considered a limitation that leads to poor pharmacokinetics, such as a short half-life in vivo [160]. Strategies based on the reconstruction of Fc functions for nanobodies have been developed to solve this challenge [159]. Therefore, the fusion between nanobody (VHH) and Fc was investigated to restore the biological functions of Fc, and laboratory studies indicated that this fusion protein could target cancer cells through CDC [159, 161]. In this regard, a study was conducted on an anti-EGFR nanobody for C-terminal modification with dinitrophenyl (DNP) hapten to restore Fc functions indirectly. This research showed that the nanobody-DNP compounds obtained have high affinity with human EGFR expressed on target cancer cells. These results may be because nanobodies have low immunogenicity, and anti-DNP antibodies are natural in the human system [159, 162]. In addition, the conjugates may prevent the rapid renal clearance of nanobodies and the lysosomal degradative effect through the classical recycling mechanism due to the formation of large immune complexes with DNP antibodies. Considering the abundance of anti-DNP antibodies in the human blood system, this method can be a practical approach to restore Fc functions and develop nanobody-based cancer immunotherapy [159]. Another way to use Fc in improving the function of nanobodies is to use the neonatal Fc receptor (FcRn) [163]. FcRn is a protein encoded by humans’ FCGRT gene and structurally similar to the MHC class I molecule. This protein is also related to beta-2 microglobulin [164, 165]. Observations suggest that FcRn contributes to tissue drug accumulation and may be a valuable target for improving tumor drug and nanoparticle penetration. Therefore, the engineering capabilities of transferring FcRn to nanobodies and its effect on tumor penetration are under investigation [163]. According to one report, FcRn can enable the non-invasive delivery of protein-containing nanoparticles and a favorable therapeutic effect in lung and intestinal diseases. Furthermore, these FcRn-targeted nanoparticles may also help make oral or intranasal vaccines and improve the efficacy of topical drugs. Hence, it is essential to understand the cellular fate of Fc-modified nanoparticles delivered to intestinal or lung tissue and their potential to induce immunity in vivo [163, 166]. However, care must be taken when designing multivalent nanoparticle systems targeting FcRn because FcRn sometimes plays a dual role in the transport of immune complexes [167].

It should be noted that Fc-fused nanobodies may have the same problems as mAb. For example, all allotypes of the Fc segment that are potentially immunogenic stimulate anti-Fc antibodies, resulting in an adverse immune response. Therefore, restoring the function of Fc nanobodies for cancer treatment should be without the disadvantages mentioned above [159].

### Enhancement of anti-tumor effects of CD8^+^ cells by anti-CTLA-4 nanobodies

Nanobodies and their engineering can effectively increase CD8^+^ T cells and treat cancer; the role of siCTLA-4-NPs in increasing the efficiency of CD8^+^ T cells [152]. Among cancer immunotherapy methods, tumor cytotoxic T lymphocyte (CTLs) treatment has attracted much attention [168]. However, their marginal efficiency in killing tumor cells jeopardizes their utility, and it is necessary to employ other ways to improve the effects of immunotherapy. Based on the findings, increasing the quality and number of adaptive T cells is a reliable way to improve therapeutic effects [169]. As previously mentioned, CTLA-4 can competitively bind to B7 and prevent further activation of T cells [170]. Therefore, to deal with the inhibitory effect of CTLA-4 on T cells, a special nanobody called CTLA-4 Nb16 was designed. This molecule was used to disrupt CTLA-4 signaling and overcome the negative stimulation of T cells [128]. Based on a study in hepatocellular carcinoma (HCC), upon CTLA-4 Nb16 stimulation, dendritic cell/hepatocellular carcinoma fusion cells (DC/HepG2-FCs) increased autologous CD8^+^ T cell proliferation and IFN-γ production in vitro. This therapeutic approach resulted in the increased killing of tumor cells and significantly suppressed tumor growth in murine NOD/SCID hepatocarcinoma xenograft models [169]. These findings show that specific CTLs induced with DC/tumor cells show superior anti-tumor effects in response to nanobody stimulation. As a result, this method can be considered a potentially valuable tool for achieving targeted immunotherapy in cancer patients [169].

### Delivery of anti-CTLA-4 Nanobodies and the Role of Engineered Probiotics

Most of the studies refer to the delivery of nanobodies intravenously. Because the size of nanobodies is small, it exposes them to rapid renal clearance. Therefore, an approach to modify nanobodies to increase their half-life in serum has been considered. Nanobody secretory carriers can solve this problem with continuous and local delivery [86]. For example, genetically engineered probiotics can be used for the local delivery of checkpoint-blocking nanobodies [171, 172]. With the help of these probiotics, anti-PD-L1 and anti-CTLA-4 nanobodies can be used intratumorally. This method results in an enhanced systemic immune response and synergistic effects of granulocyte-macrophage CSF (GM-CSF) [86, 171]. *E. coli* Nissle (EcN) 1917 is a bioengineered bacteria carrier of anti-PD-L1 and anti-CTLA-4 nanobodies. This bacterium strain is a gram-negative microorganism with probiotic properties used to treat cancer [172, 173]. In addition, liposomes, polymersomes, immunotoxins, micelles, albumin-based nanoparticles (NANAPs), and nanobody-drug conjugates (NDCs) are nanobody-based carriers in treating various cancers. These carriers provide different drug delivery potentials of nanobodies [174].

Oral delivery of nanobodies is another method for which acceptable potentials have been found [175]. In addition to intravenous and oral delivery of nanobodies, viral vectors can also be used to code targeted nanobodies inside the tumor. However, it should be noted that this method needs more studies in the body. One of the methods used is the bacterial type III protein secretion system (T3SS) to deliver nanobodies to tumor cells, which has successfully resulted in anti-amylase, anti-EGFP, and anti-GFP nanobodies in vitro and in vivo. This method injects nanobodies into the cytoplasm by a molecular syringe [86, 176, 177]. It is essential to mention that one of the main obstacles to the potential of T3SS needs to be clarified targeting. However, this problem can be overcome by conjugating the nanobodies to the bacterial surface [86].

In addition to treatment, nanobodies can have diagnostic applications and be used in tumor imaging [178]. Intravenous delivery of probes based on nanobodies can be used for imaging applications. Of course, this is not true about brain tumors because the BBB significantly hinders their absorption. However, a recent study showed that intra-arterial administration of nanobody imaging probes dramatically increased delivery regardless of BBB status. This could be a potential strategy to bypass BBB restrictions [86, 179].

## Drug therapies based on CTLA-4

**Fig. 6:**
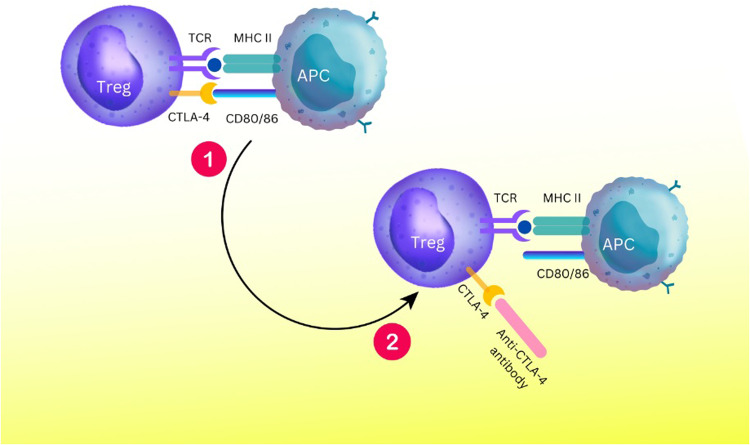
Functional role of anti-CTLA-4 antibodies. Drugs can increase the activity of Treg cells with the help of anti-CTLA-4 antibodies and by inhibiting the CTLA-4 molecule.

The utilization of CTLA-4 immunoglobulins (CTLA-4 Ig) has been expanded to block the binding to the B7 family of molecules to inhibit T cell proliferation [60]. Furthermore, using antibodies to control the CTLA-4/CD28 pathway is envisaged to treat cancers and autoimmune diseases (Fig. 6) [180]. Abatacept was the first drug in this field. This drug was approved in 2005 for the treatment of rheumatoid arthritis. The US Food and Drug Administration (FDA) later approved it for treating active psoriatic arthritis [181]. However, CTLA-4 Ig is not practical for the treatment of disorders such as multiple sclerosis (MS) and ulcerative colitis (UC) [182, 183]. Ipilimumab, a type of human monoclonal antibody, is another drug in this sector that acquired approval from the FDA in 2011 to treat patients with metastatic melanoma [139, 184]. The clinical trial results indicate that utilizing ipilimumab at 1 and 3 mg/kg dosages effectively interrupted CTLA-4 signaling, resulting in anti-tumor activity in patients with B cell lymphoma [185]. Tremelimumab is another medicine studied in this field, and although its trial results did not equal those of ipilimumab, it has shown promising efficacy in melanoma patients [186–188]. This drug avoids the interactions between CTLA-4 and CD28 [189]. Tremelimumab has also been used with other immune-suppressing drugs to study the possibilities of treating different cancers [5]. These drugs are based on anti-CTLA-4 antibodies, and it is necessary to adopt approaches that use similar molecular mechanisms and nanobody-based anti-CTLA-4 drugs to treat diseases, especially types of cancers.

## Challenges and limitations of utilization of nanobodies

There are still some significant limitations and disadvantages to nanobodies, which makes them limited to a relatively small number of applications. For one, Nbs are obtained from camelids and sharks, while mAbs can be obtained from mice [190]. This is a significant drawback since obtaining Nbs from immunized camelids is more costly than the mAbs that routinely used mice produce. Another hitch of utilizing camelids is related to their housing. After the immunization round, these animals will not be sacrificed and must be provided with continuing shelter for their lives. However, some efforts have been made to perform the immunization at a safe distance from the laboratories and to analyze cDNAs and phage libraries in the laboratory [74]. Also, studies have shown that camelids can tolerate restricted modifications because merely a single domain in the VHH domain composed of approximately 110 amino acids is accessible, so every residue in the domain faces loads of weight [191].

Although the small size of Nbs is beneficial in many cases, it is considered a disadvantageous property in some approaches. Since the threshold of glomerular filtration is about 65 kDa, the filtration of Nbs is swiftly done. This can be a significant drawback in therapies requiring long-term antibody circulation. Furthermore, their lower half-life in the blood can result in a lower chance of hitting the target [192, 193]. As described earlier, Nbs can be rapidly cleared through the kidneys. If they are conjugated with toxic agents, there will be a risk of renal toxicities due to their accumulation [9, 194]. Another obstacle in applying Nbs is using biohazardous means such as bacteriophages to produce them [195]. This can cause extra expense as there is an urge for safe disposal. Also, Nbs are not considered rapidly biodegradable materials, creating concerns about their effect on agriculture [196, 197].

Furthermore, the inability of Nbs to cross the BBB effectively acts as a double sword. It can be an advantage when the area of the target is outside the nervous system, and the off-target side effects will be lower. However, when there is a need to target the central nervous system, this can be a hitch [198]. However, there has been much effort to address the previously described issues. Nevertheless, questions still need to be answered, and further research is needed to shed light on applying Nbs to different diseases.

## Discussion

Among cancer treatments, immunotherapy is an emerging and attractive field whose primary goal is to use the body’s immune system to identify and destroy tumor cells. Various types of immunotherapy are being researched and developed in multiple preclinical and clinical stages. Meanwhile, checkpoint inhibition is a new approach in cancer immunotherapy that is rapidly advancing. This method can be adjuvant or alternative to traditional cancer treatments [20]. When a T cell is activated, it prevents over-activation by regulating co-inhibitory molecules such as CTLA-4 and PD-1. These co-inhibitory molecules, intrinsic molecules of the cell, inhibit mTOR function through PP2A or SHP-2 signaling, respectively [199]. mTOR is a critical molecule and a member of the PI3-kinase family. This evolutionarily conserved molecule is vital in integrating environmental signals, including extracellular growth and survival, cell activation status, amino acids, oxygen availability, and intracellular ATP concentration. In addition to the above, the increasing role of mTOR in the activation, differentiation, metabolism, and function of T cells has been established [200]. Immune checkpoints are negative regulators of immune activation that limit anti-tumor responses. This leads to long-lasting tumor responses in cancer patients [201]. Also, T cells in the tumor microenvironment (TME) upregulate the expression of checkpoint molecules such as cytotoxic T lymphocyte-associated protein 4 (CTLA-4) [20, 199]. In other words, checkpoint inhibitors block checkpoint molecules and allow the adaptive immune system to respond to tumors. Therefore, applying methods that generate tumor-specific T cells for the effectiveness of checkpoint inhibition can be a great potential for changing cancer treatment methods [202, 203]. Among the immunotherapy methods, using nanobodies is very effective in facilitating the presentation of tumor antigens and inducing the abscopal effect. This method increases the synergistic impact between radiotherapy and checkpoint immunotherapy [152]. Nanobodies are essential for stable system-producing biological drugs for diagnosis and therapeutic interventions [204]. As a result, nanobody engineering has received attention in recent years. For example, one approach is engineering the spore-forming bacterium *Bacillus subtilis* to secrete Nbs that target specific molecules in mammalian cells [205]. Nanobodies, unlike mAbs, lack the Fc region, so they cannot directly initiate an Fc-mediated immune response. Hence, the use of Fc-conjugated nanobodies was investigated. This strategy can help limit the size of nanobodies against rapid renal clearance and increase their half-life [206, 207].

Also, other combined treatment approaches can be practical, such as using Nb-siRNA or CAR-T cell-based nanobodies [155, 208]. CAR-T cell therapy as an independent method has achieved stunning therapeutic successes. This method increases the reactivity of their target tumor by blocking the immune checkpoint. Although PD-1 has been the leading candidate of this method, outstanding results can be achieved by combined checkpoint blockade of multiple inhibitory pathways such as PD-1 and CTLA-4 [208]. Of course, it should be noted that according to the mechanisms in the TME, the effectiveness of CAR-T cell treatment is associated with limitations such as physical barriers for the effective penetration of CAR-T cells and types of immunosuppressive cells. Therefore, by understanding the improvement of the inherent resistance of TME, new therapeutic strategies and complex designs of CAR are proposed [141]. These strategies include using new methods of T cell engineering and gene editing to enhance the delivery of CAR-T cells to the TME site, counteract suppressive mechanisms, and enhance anti-tumor response. As a result, overcoming TME obstacles for CAR-T treatments requires synergistic approaches with other treatment methods [141]. Since Treg cells in the TME are a barrier to anti-tumor immune mechanisms and T cell priming, reducing the function of Tregs by potentiating anti-CTLA-4 mediators may be an essential strategy for the development of next-generation anti-CTLA-4 immunotherapy [209].

The studies have proven the diagnostic application of anti-CTLA-4 nanobodies in pet imaging and therapeutic applications in cytolytic cell therapy and immune restoration [89, 210]. Furthermore, these results can be promising for cancer treatments based on anti-checkpoint nanobodies on a large scale in the future.

## Conclusion and future perspective

Tumor immunotherapy has been investigated worldwide due to its potent properties and long-term therapeutic effect. In addition, targeted treatments and immunotherapy based on nanobodies have recently received much attention. This interest originates from some structural features of nanobodies, including small size, hydrophilic site, ease of labeling, availability, high flexibility and resistance, low viscosity, and the ability to engineer multi-purpose structures. Due to these advantages, it is possible to non-invasively image malignant cells and design specific treatments to target tumor cell antigens, immune cells, and tumor environment proteins by nanobodies so that they can be used as diagnostic and therapeutic agents in immuno-oncology. Therefore, the research and development of nanobody-based drugs in the preclinical and clinical stages of cancer treatment are under investigation.

Nanobodies have shown good therapeutic results despite the lack of practical function mediated by Fc. However, the production of modified nanobodies with Fc-receptors has been done to deal with some limitations, including rapid clearance by the kidneys due to their small size. Among cancer immunotherapy methods, considerable progress has been made in treatments based on anti-immune checkpoint antibodies, and FDA-approved inhibitors, including CTLA-4, are widely used in various malignancies. However, their clinical application has faced difficulties due to the limited response of safe investigational drugs.

With the prominence of nanotechnology in biomedical studies and the application of this science in inhibiting the immune checkpoint, it is possible to overcome the challenges ahead and achieve brilliant results with the help of combined treatments. Based on these cases, anti-CTLA-4 nanobodies can be investigated in clinical phases. If the clinical studies are successful, this method can be used for targeted anti-tumor treatments in a wide range.

### Supplementary information

checklist
